# Forelimb muscle activation patterns in American alligators: Insights into the evolution of limb posture and powered flight in archosaurs

**DOI:** 10.1111/joa.14011

**Published:** 2024-01-19

**Authors:** Masaya Iijima, Christopher J. Mayerl, V. David Munteanu, Richard W. Blob

**Affiliations:** ^1^ Structure and Motion Lab, Department of Comparative Biomedical Sciences The Royal Veterinary College Hertfordshire UK; ^2^ Nagoya University Museum Nagoya Japan; ^3^ Department of Biological Sciences Northern Arizona University Flagstaff Arizona USA; ^4^ Department of Biological Sciences Clemson University Clemson South Carolina USA

**Keywords:** biomechanics, Crocodylia, electromyography, locomotion, reptile

## Abstract

The evolution of archosaurs provides an important context for understanding the mechanisms behind major functional transformations in vertebrates, such as shifts from sprawling to erect limb posture and the acquisition of powered flight. While comparative anatomy and ichnology of extinct archosaurs have offered insights into musculoskeletal and gait changes associated with locomotor transitions, reconstructing the evolution of motor control requires data from extant species. However, the scarcity of electromyography (EMG) data from the forelimb, especially of crocodylians, has hindered understanding of neuromuscular evolution in archosaurs. Here, we present EMG data for nine forelimb muscles from American alligators during terrestrial locomotion. Our aim was to investigate the modulation of motor control across different limb postures and examine variations in motor control across phylogeny and locomotor modes. Among the nine muscles examined, m. pectoralis, the largest forelimb muscle and primary shoulder adductor, exhibited significantly smaller mean EMG amplitudes for steps in which the shoulder was more adducted (i.e., upright). This suggests that using a more adducted limb posture helps to reduce forelimb muscle force and work during stance. As larger alligators use a more adducted shoulder and hip posture, the sprawling to erect postural transition that occurred in the Triassic could be either the cause or consequence of the evolution of larger body size in archosaurs. Comparisons of EMG burst phases among tetrapods revealed that a bird and turtle, which have experienced major musculoskeletal transformations, displayed distinctive burst phases in comparison to those from an alligator and lizard. These results support the notion that major shifts in body plan and locomotor modes among sauropsid lineages were associated with significant changes in muscle activation patterns.

## Graphical abstract

Electromyographic data from the forelimb muscles of American alligators indicate that alligators modulate the activity patterns of a primary shoulder adductor muscle across a range of limb postures. Among sauropsids, forelimb muscle burst phases from birds and turtles are distinct compared to those of alligators and lizards, suggesting that muscle activation patterns were influenced by major changes in body plan and locomotor modes.

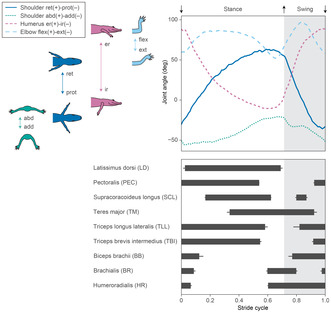

## 1 INTRODUCTION

Archosauria is a group of tetrapods that includes the most recent common ancestor of crocodylians and birds and all its descendants (Cope, 1869; Nesbitt, 2011). Since their origin around 250 Ma in the earliest Mesozoic, archosaurs have undergone remarkable diversification across land, water, and air (Brusatte et al., 2008; Lloyd et al., 2008; Nesbitt, 2011; Sereno, 1997; Wilberg et al., 2019). They have also demonstrated resilience and adaptability in the face of mass extinctions and climate change throughout the Mesozoic and Cenozoic, currently consisting of around 30 crocodylian and 10,000 bird species (Barrowclough et al., 2016; Benton, 2021; Brusatte et al., 2008, 2015; Grigg & Kirshner, 2015; Mannion et al., 2015; Markwick, 1998).

Archosaur evolution is characterized by multiple major locomotor transitions during the Mesozoic. First, a shift from a more abducted ‘sprawling’ limb posture to a more adducted ‘erect’ limb posture occurred during the Triassic (Charig, 1972). Multiple archosaur subgroups underwent skeletal transformations associated with such postural shifts, including fenestration of the acetabulum, development of a strong supraacetabular crest, and medial deflection of the femoral head (Bonaparte, 1984; Carrano, 2000; Charig, 1972; Egawa et al., 2022; Griffin et al., 2022; Hutchinson, 2001a, 2001b, 2006; Hutchinson & Gatesy, 2000; Iijima & Kobayashi, 2014; Parrish, 1986). Although this anatomical evidence suggested that the transition to erect limb posture may have occurred gradually, fossil trackways suggest that the shift toward more erect limb posture might have occurred rather abruptly in the Early Triassic (Kubo & Benton, 2009).

Second, powered flight evolved in two archosaur lineages, pterosaurs and paravians, although they exhibit different wing structures and launching styles (Habib, 2008; Martin‐Silverstone et al., 2020; Palmer, 2011; Witton & Habib, 2010). For paravians, the evolution of an erect limb posture and bipedality, which freed the forelimb from the terrestrial locomotor module, paved the way for the acquisition and diversification of their flight capabilities from the Late Jurassic onwards (Charig, 1972; Gatesy & Dial, 1996; Padian & Chiappe, 1998; Pei et al., 2020; Sullivan, 2015).

To understand these major locomotor transitions, the soft tissue anatomy and physiology of extant archosaurs, crocodylians and birds, provide critical data. Previous studies on crocodylian locomotion have focused on their limb kinematics, forces, and bone loading, revealing their distinct ‘low walk’ that differs kinematically from the sprawling locomotion in lizards, and an unexpected increase in hindlimb bone loading associated with the use of more upright limb posture (Blob & Biewener, 1999, 2001; Brinkman, 1980; Gatesy, 1991; Iijima et al., 2021; Manafzadeh et al., 2021; Reilly & Elias, 1998). Moreover, comparisons between crocodylian and bird locomotion, as well as observations of fossil taxa, revealed structural and mechanical changes related the evolution of powered flight, such as an anterior shift in the center of mass and a transition from hip‐ to knee‐based hindlimb kinematics in theropod dinosaurs (Allen et al., 2013, 2021; Gatesy, 1990, 1999; Hutchinson & Gatesy, 2000).

In addition to soft tissue anatomy, muscle activation patterns can only be measured in living animals through electromyography (EMG), which provides insights into the timing, intensity, and frequency of myoelectric signals across a range of speed and locomotor modes employed by an animal (Blob et al., 2008; Cappellini et al., 2006; Foster & Higham, 2014; Gatesy, 1994, 1997; Gorvet et al., 2020; Loeb & Gans, 1986; Mayerl et al., 2017; Rivera & Blob, 2010; von Tscharner, 2000; Wakeling et al., 2002). With regard to shifts in posture, a comparison of hindlimb muscle activation in American alligators showed increases in the activation of knee and ankle extensors when assuming a more upright limb posture (Reilly & Blob, 2003). This finding, which may appear counterintuitive, is nevertheless consistent with skeletal loading patterns across various hindlimb postures, suggesting that the use of a more upright limb posture may not reduce antigravity muscle activation and bone loading in alligators (Blob & Biewener, 1999). However, it is unclear whether the patterns of posture‐dependent modulation of muscle activation observed in the alligator hindlimb can be used to predict modulation patterns for forelimb muscles as well. Specifically, the mechanism behind the reduced activation of hindlimb antigravity muscles during a more upright walk in alligators is associated with their unique foot geometry and the presence of biarticular muscles crossing the ankle and knee joints (Reilly & Blob, 2003). This mechanism may not be directly applicable for the alligator forelimb with a short manus.

Beyond intraspecific observations, comparisons of EMG data across taxa can provide further insights into the evolution of muscle activation patterns. Previous comparisons of forelimb and hindlimb EMG data among tetrapods have revealed broadly similar activation timings of homologous muscles, with minor variations attributed to different limb kinematics (Ashley‐Ross, 1995; Cuff et al., 2019; Dial et al., 1991; Pierce et al., 2020; Rivera & Blob, 2010, 2013). However, there is relatively little comparative EMG data available for forelimb muscles in tetrapods. Specifically, in the case of crocodylians, only m. pectoralis has been examined for its EMG activity (Cuff et al., 2019). This limited dataset hinders our ability to understand the changes in muscle activation timings associated with the evolution of powered flight in paravians. Consequently, it remains unclear whether forelimb muscle activation patterns in archosaurs are conserved across taxa, or vary depending on their body plan, locomotor modes, and habitat.

In this study, we report EMG data for nine forelimb muscles from American alligators. Our primary objectives were to (1) investigate modulation of forelimb muscle activation across different limb postures, and (2) examine variations in forelimb muscle activation among tetrapods, with a particular focus on those associated with different locomotor modes (e.g., terrestrial vs. aerial locomotion). Our specific predictions are: (1) a more adducted forelimb posture is correlated with reduced activation of shoulder adduction muscles, which would reduce stance phase force and work, and (2) forelimb motor patterns have been conserved across tetrapods, irrespective of different locomotor modes. Through our comparisons, this study contributes to the understanding of the neuromuscular changes related to major locomotor transitions in vertebrate evolutionary history.

## 2 MATERIALS AND METHODS

### 2.1 Animals and target muscles

Three juvenile American alligators, *Alligator mississippiensis* (Daudin, 1802), identified as al13, al14, and al15, with total length of 0.8–0.9 m and weight of 1.8–1.9 kg, were used in this study. These animals were captured from the wild by staff biologists and technicians of Louisiana Department of Wildlife and Fisheries alligator program and then transported by car to Clemson University, SC, USA. The alligators were housed in a greenhouse vivarium facility with ambient lighting and daily temperature range of 23–38°C for five months prior to the start of the experiment. During this time, they were fed commercial pellets (Mazuri crocodilian diet, small) twice per week.

Nine pectoral and upper arm muscles were recorded for their activation patterns among 13 targeted muscles. The nine successfully recorded muscles (with predicted actions in parentheses) included: m. latissimus dorsi (LD: shoulder extensor and humeral retractor; shoulder abductor and humeral elevator), m. pectoralis (PEC: shoulder adductor and humeral depressor), m. supracoracoideus longus (SCL: shoulder flexor and humeral protractor), m. teres major (TM: shoulder abductor and humerus elevator), m. triceps longus lateralis (TLL: elbow extensor), m. triceps brevis intermedius (TBI: elbow extensor), m. biceps brachii (BB: elbow flexor), m. brachialis (BR: elbow flexor), and m. humeroradialis (HR: elbow flexor) (Allen et al., 2015; Meers, 2003: Figure 1). Among the 13 muscles initially targeted, m. coracobrachialis brevis ventralis (CBV), m. subscapularis (SS), m. deltoideus scapularis (DS), and m. scapulohumeralis caudalis (SHC) (Allen et al., 2015; Meers, 2003) could not be recorded for their muscle activities, despite our extensive efforts to implant multiple electrodes in multiple individuals. This was due to hindrances caused by superficial muscles, wire misplacements, and cable disconnection after the surgery. Description of the successfully recorded muscles, including their origins, insertions, spatial relationships with neighboring muscles, and estimated actions can be found in previous works (Allen et al., 2015; Meers, 2003). Although the three heads of mm supracoracoideus complex (namely mm. supracoracoideus longus, intermedius, and brevis: Meers, 2003) can be difficult to isolate, post‐experiment dissections (see below) verified that all electrodes placed in the muscle complex were in the most superficial belly, m. supracoracoideus longus.

**FIGURE 1:**
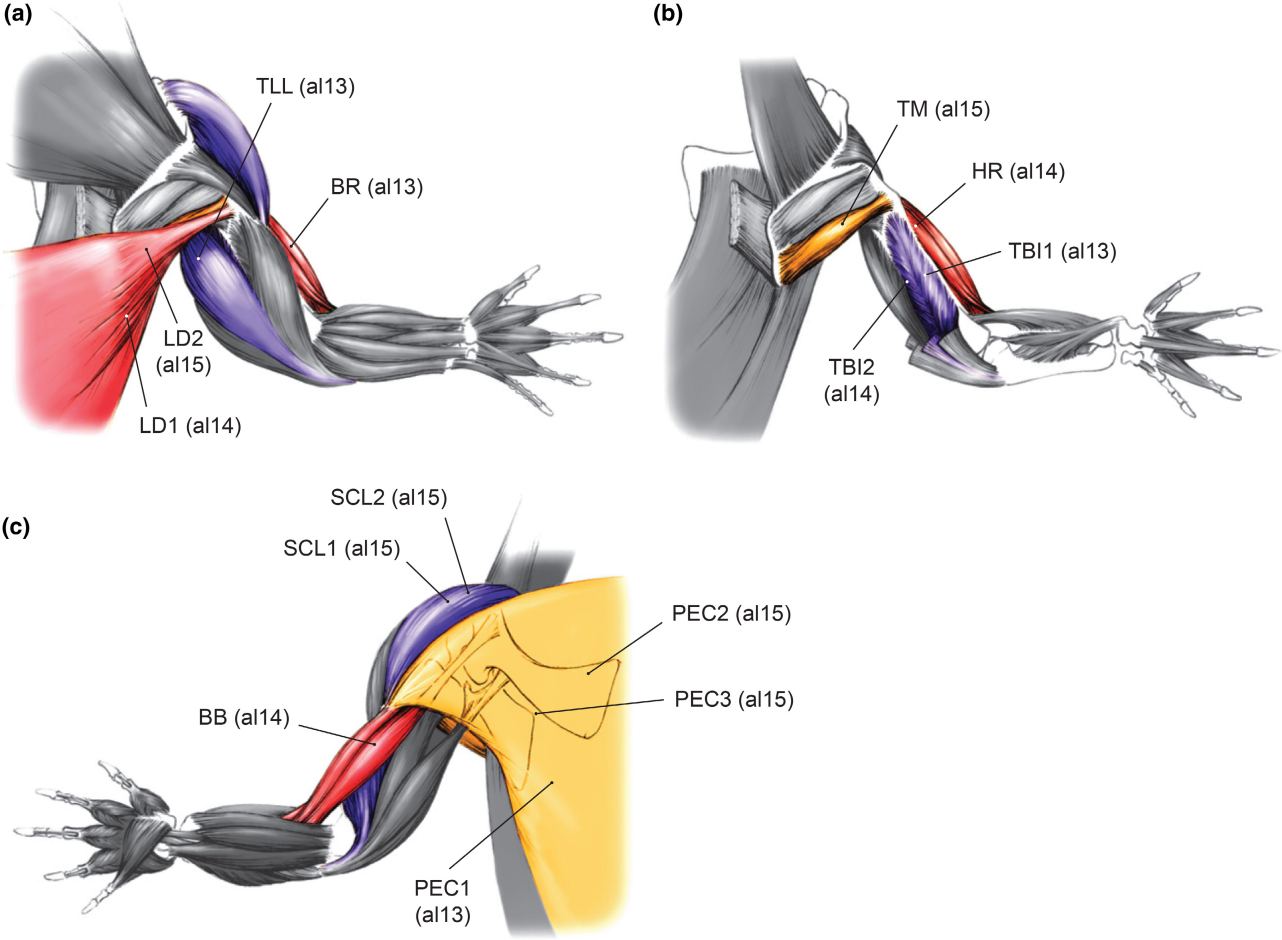
Forelimb muscles of American alligators from which EMG data were collected in (a) superficial dorsal, (b) deep dorsal, and (c) ventral views. Electrode placements are indicated by lines. Drawings of forelimb anatomy were modified from Allen et al. (2015). Muscle abbreviations: BB, m. biceps brachii; BR, m. brachialis; HR, m. humeroradialis; LD, m. latissimus dorsi; PEC, m. pectoralis; SCL, m. supracoracoideus longus; TBI, m. triceps brevis intermedius; TLL, m. triceps longus lateralis; TM, m. teres major. Labels in parentheses indicate animals (al13, al14, or al15) from which EMG data were recorded.

### 2.2 Electrode implantation

Alligators were sedated with intramuscular injections of ketamine (10 mg kg^−1^). Insulated stainless steel bipolar fine wire electrodes (50 μm diameter; California Fine Wire, Grover Beach, CA) were implanted into target muscles using 26‐gauge hypodermic needles. Twelve electrodes were used for each alligator, with multiple electrodes being placed in each muscle to maximize successful EMG recording. The electrode wires were glued together to form two bundles, which were connected to shielded cables through micro connectors. These electrode wires and cables were secured anterior to the pelvis using self‐adhesive Vet Wrap bandages, providing some slack for the electrode bundles to accommodate movement. Alligators fully recovered from sedation a few hours after the implantations and were ready to participate in trials. Out of 12 electrodes implanted in each animal, an average of 4–5 electrode channels produced clean signals, resulting in a total of 14 successful channels across three individuals. After data collection, animals were euthanized through intraperitoneal injection of sodium pentobarbital euthanasia solution (120 mg kg^−1^), and electrode placements were confirmed by dissection. Implantation and experimental procedures were approved by Clemson University IACUC (AUP 2019‐037).

### 2.3 Speed and kinematic measurements

After the EMG wire and cable connectivity were confirmed in LabVIEW v.6.1 (National Instruments, Austin, TX), the alligators were encouraged to walk on a treadmill, using light tapping on the tail for stimulation when necessary. Treadmill speed was adjusted to match the natural walking speed for each alligator. Each trial was concluded after an alligator walked consistently at the steady treadmill speed for eight seconds, which represented the maximum recording time for EMG data in our recording system. Two digitally synchronized Phantom v.5.1 high speed cameras (Vision Research, Wayne, NJ, USA) filmed the walking steps from dorsal and dorsolateral views at 100 Hz. 3D camera calibration and coordinate digitization were performed using DLTcal8 and DLTdv8 (Hedrick, 2008) in MATLAB R2022B (MathWorks, Natick, MA). To synchronize the EMG data and video footage, a trigger simultaneously sent a 1.5 V square‐wave pulse to EMG signal channels and a light pulse to the video. Walking speed was calculated using white dots painted at 10 cm increments on the treadmill belt. These speed measurements were then converted to dimensionless speed = absolute speed/(*g h*)^1/2^, where *g* is gravitational acceleration (= 9.81 m s^−2^), and *h* is extended hindlimb length from the hip to ankle.

To analyze limb kinematics, we digitized 3D joint coordinates along the trunk midline (pectoral joint medial to the shoulder joint, and the thoracic joint halfway between the pectoral joint and mid‐trunk) and forelimb (shoulder, elbow, wrist, and metacarpophalangeal joints), using white markers painted on each joint. Joint angles were quantified using the skin marker‐based joint coordinate system (JSC) described by Iijima et al. (2021, 2023). Briefly, three rotational degrees of freedom (RDF) for the shoulder [retraction(+)‐protraction(−), abduction(+)‐adduction(−), and external(+)‐internal(−) long‐axis rotation, in this rotation order], and one RDF for the elbow and wrist [flexion(+)‐extension(−)] were considered. Joint rotation around each axis was calculated based on the reference pose where the forelimb is laterally splayed out (Iijima et al., 2021, 2023). As detailed kinematic description was not the primary focus of this study, we chose to digitize only four entire strides in one representative trial (al13e04) to visualize joint angle profiles across a stride. Joint angles were resampled at 1% increments to align with mean stance and swing ratios across all strides (0.71 stance and 0.29 swing within a stride cycle). To quantify limb posture, the shoulder adduction angle was measured at mid‐stance in all available strides and trials. Mid‐stance was identified as the video frame where the shoulder and metacarpophalangeal joints are vertically aligned in the lateral view. Although the shoulder adduction angle changes throughout the stance phase, mid‐stance is a typical point at which overall characterizations of steps are compared, and mid‐stance joint angles should sufficiently represent differences in joint angles across steps (Blob & Biewener, 1999).

### 2.4 EMG recording and processing

During the locomotor trials, EMG signals were amplified ~10,000 times, with minor adjustment of the signal amplification made for each electrode channel, using a Grass 15LT Physiodata Amplifier System (Grass Instrument, West Warwick, RI). Raw EMG signals were subjected to a 30–6000 Hz bandpass filter, and then sampled at 5000 Hz using LabVIEW. EMG profiles were manually inspected to identify any crosstalk from neighboring muscles, and channels potentially affected by crosstalk were removed from the dataset. Digital EMG signals were further processed by applying a 10–500 Hz 2nd order Butterworth bandpass filter, primarily to remove high frequency noise. Subsequently, EMG signals were rectified, and a 10 Hz 2nd order Butterworth lowpass filter was applied to capture the ‘envelope’ of the EMG signals. Finally, the signals were downsampled to 500 Hz to reduce processing time. Digital filtering was performed using the R package signal (R Core Team, 2022; Signal developers, 2013).

To determine onset/offset times of muscle activities, we employed a randomization method to select a signal threshold to discriminate between the muscle signals and background noise (Mayerl et al., 2022; Thexton, 1996). Briefly, a series of equally spaced signal thresholds were applied to the EMG envelope and numbers of ‘runs’ that crossed these thresholds were counted. The same procedure was then repeated for the signals that were randomly sampled without replacement. Finally, the optimal threshold with the maximum difference in the counts of ‘runs’ between the original and randomized data was identified (Thexton, 1996). In this study, we used 50 equally spaced thresholds (2% increment within the observed signal range) for the randomization thresholding method. Muscle onset/offset times determined using the optimal threshold were manually examined and adjusted if necessary (i.e., grouping multiple isolated bursts into a single burst). Onset/offset times were normalized to mean stance and swing ratios across all strides (0.71 stance and 0.29 swing within a stride cycle) and expressed as a relative stride time ranging from 0 to 1. Following the determination of onset/offset times, the mean EMG amplitude was calculated as the area under the curve of the rectified EMG signal divided by the burst duration (μV), and the burst duration was normalized to a relative stride time ranging from 0 to 1.

### 2.5 Analysis

To test the relationship between EMG characteristics and forelimb posture, multiple linear regressions were performed with mean EMG amplitude or normalized burst duration as the response, shoulder adduction angle and dimensionless speed as predictors, and the interaction term, using the R package pequod (Mirisola & Seta, 2016; R Core Team, 2022). We examined the interaction between two predictors, shoulder adduction angle and dimensionless speed, because speed may influence limb posture (Gatesy & Biewener, 1991; Irschick & Jayne, 2000; Reilly & Elias, 1998). Predictors were centered around their means to reduce multicollinearity. Regression analyses were conducted for each muscle from each individual, excluding EMG recordings from a single trial with no variation in walking speed. A pooled analysis that combines EMG data from multiple electrodes for each muscle was not conducted. This is due to the challenges in comparing EMG characteristics across electrodes, which are influenced by differences in electrode placements, cable connectivity, and signal amplification. For muscles characterized by biphasic bursts, the primary burst with higher amplitude and longer duration was used for the analysis. Shorter second bursts, where observed, generally had lower amplitudes than the primary bursts (see results).

Muscle activation patterns were compared among tetrapods with a special focus on the lineage leading to archosaurs. Forelimb muscle onset/offset timings during aerial or terrestrial locomotion were obtained from literature for a passeriform bird (*Sturnus vulgaris*: Dial et al., 1991), an emydid turtle (*Trachemys scripta*: Rivera & Blob, 2010), a varanid lizard (*Varanus exanthematicus*: Jenkins & Goslow, 1983), and a urodele amphibian (*Salamandra salamandra*: Pierce et al., 2020). EMG activities of six forelimb muscles homologous to those in American alligators, including LD, PEC, SCL, TLL, TBI, and BB, were recorded in these species. With regard to LD from *Varanus*, EMG recordings from middle and posterior parts of the muscle were adopted (Jenkins & Goslow, 1983). For urodeles, we acknowledge that EMG activities of several forelimb muscles during terrestrial locomotion were recorded in the northern crested newt (*Triturus cristatus*: Székely et al., 1969). However, due to the difficulty in extracting muscle onset/offset timings from their raw EMG recordings, they were not included in the analysis. Forelimb muscle homologies were adopted from Diogo et al. (2018) and Smith‐Paredes et al. (2022). To facilitate visual comparison of muscle activation across taxa, onset/offset timings were arbitrarily normalized to 0.7 stance (upstroke) and 0.3 swing (downstroke) within a stride (stroke) cycle to create bar plots. The normalized stance to stride ratio of 0.7 should be reasonable, given that it ranges between 0.54 and 0.79 in four taxa compared (Dial et al., 1991; Jenkins & Goslow, 1983; Pierce et al., 2020; Rivera & Blob, 2010).

To quantitatively compare EMG activities of multiple muscles across taxa, pairwise distances of muscle activation phases were computed. First, muscle onset/offset timings were normalized to 0.5 stance (downstroke) and 0.5 swing (upstroke) within a stride (stroke) cycle, where 0 and 1 represent touchdown (upstroke‐downstroke transition) and 0.5 represents takeoff (downstroke‐upstroke transition). The first and second bursts of m. triceps (scapular head) for *Trachemys* were grouped together because the duration of the short second burst was at least three times longer than either of the non‐burst durations. Additionally, short second bursts of m. supracoracoideus for *Alligator* and *Varanus* and m. biceps brachii for *Sturnus* were removed to make phase comparisons between the primary EMG bursts. Second, mid EMG burst phases (mod 1) were calculated as onset + (offset – onset)/2 [if offset < onset, then onset + (offset + 1 – onset)/2], such that a muscle with an onset of 0.8 and an offset of 0.4 would have a mid EMG burst phase of 0.1. These were then used to compute the pairwise phase distances in all taxon pairs for each muscle, by determining the absolute difference in each pair of mid EMG burst phases (if |difference| exceeds 0.5, then 1 – |difference|). Pairwise phase distances of each muscle were multiplied by two to place them on a 0 to 1 scale, such that a muscle with a mid EMG burst phase of 0.9 in one animal and 0.1 in another would yield a pairwise phase distance of 0.2 multiplied by 2, equaling 0.4. After inspecting the data, we decided to remove all taxon pairs with *Salamandra*, as less than three muscles were available for those pairs. Third, the overall pairwise distances of EMG burst phases among four sauropsids (*Sturnus*, *Alligator*, *Trachemys*, and *Varanus*) were computed using the maximum observable rescaled distance (MORD), which is the Euclidean distance divided by the maximum realizable distance (Lloyd, 2016), based on pairwise burst phase distances of all available muscles. The MORD method places distances on a strict 0 to 1 scale (Lloyd, 2016).

The EMG burst phase distance matrix, displaying overall pairwise EMG burst phase distances between all taxon pairs with diagonal elements all zero (see results), was used to generate a ‘phylo‐EMG space’ of six forelimb muscles [m. latissimus dorsi, m. pectoralis, m. supracoracoideus, m. triceps (scapular and humeral heads), and m. biceps brachii] from four sauropsids. The distance matrix was subjected to a principal coordinate (PCO) analysis using the R function cmdscale (*k* = number of taxa – 1; add = TRUE) (R Core Team, 2022). PCO scores of all three axes were then plotted on the phylogeny of four taxa, using the R packages phytools and rgl (Murdoch & Adler, 2023; R Core Team, 2022; Revell, 2012). The phylogenetic relationships of four taxa, particularly the placement of turtles, followed molecular evaluations (Chiari et al., 2012; Crawford et al., 2012; Kumazawa & Nishida, 1999; Zardoya & Meyer, 1998), and their divergence times followed Irisarri et al. (2017). The root length of the phylogenetic tree was set at 1 Ma.

## 3 RESULTS

**FIGURE 2:**
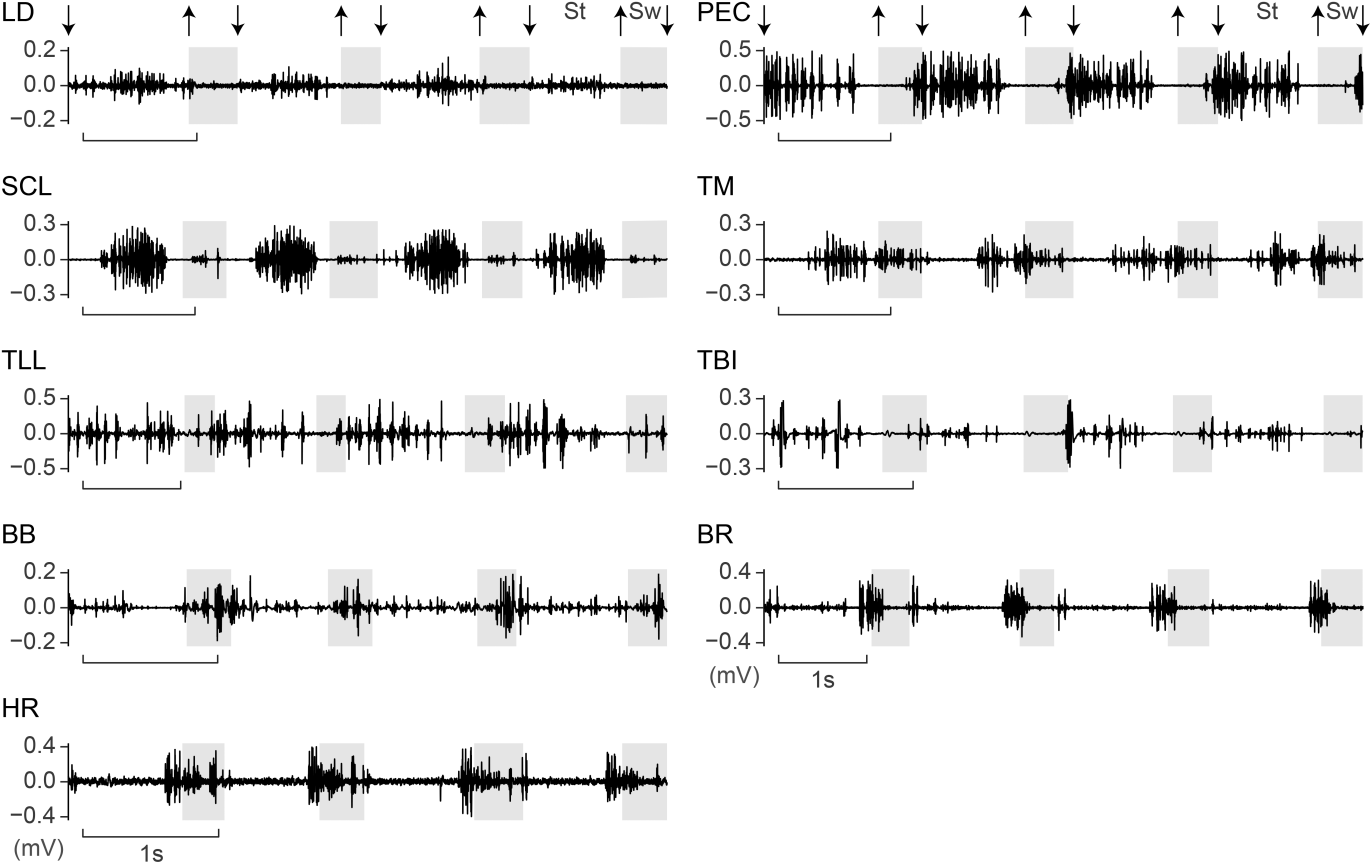
Representative filtered EMG signals for nine forelimb muscles from American alligators. Muscle abbreviations: BB, m. biceps brachii; BR, m. brachialis; HR, m. humeroradialis; LD, m. latissimus dorsi; PEC, m. pectoralis; SCL, m. supracoracoideus longus; TBI, m. triceps brevis intermedius; TLL, m. triceps longus lateralis; TM, m. teres major; St, stance; Sw, swing. Downward pointing arrows indicate placement of the manus on the ground, upward pointing arrows indicate raising of the manus off the ground, and shaded areas indicate swing phase.

EMG activities for nine forelimb muscles (LD, PEC, SCL, TM, TLL, TBI, BB, BR, and HR) were recorded for 6–118 strides from 1 to 3 electrode channels and 1–2 animals each (Table 1; Figures 1 and 2). Among nine muscles, four were recorded from multiple electrodes, and three were recorded from multiple individuals. Muscles recorded from multiple electrode channels and/or animals exhibited largely congruent burst timings. The largest mean cross‐channel and ‐individual differences in normalized burst onset and offset timings in a single muscle (0 to 1) were 0.069 for the primary burst onset of two PEC channels in al15, and 0.145 for the primary burst offset of individual LD channels in al14 and al15 (Table S1). The remaining muscles were recorded from a single electrode and individual, and caution should be taken when interpreting their EMG signals, as variations within and across individuals were not considered. However, we confirmed that the EMG characteristics of those muscles were largely consistent with the expected functions based on their anatomy.

**TABLE 1:** Summary of forelimb EMG onset and offset (mean ± standard error) normalized to a relative stride time ranging from 0 to 1 (0.71 stance and 0.29 swing within a stride cycle).

Muscle	Number of animals	Number of channels	Number of strides	Burst 1 onset	Burst 1 offset	Burst 2 onset	Burst 2 offset
Latissimus dorsi	2	2	57	0.025 ± 0.012	0.690 ± 0.013		
Pectoralis	2	3	118	0.923 ± 0.005	0.541 ± 0.003		
Supracoracoideus longus	1	2	100	0.167 ± 0.004	0.623 ± 0.003	0.799 ± 0.007	0.869 ± 0.007
Teres major	1	1	17	0.335 ± 0.019	0.922 ± 0.020		
Triceps longus lateralis	1	1	7	0.821 ± 0.042	0.582 ± 0.019		
Triceps brevis intermedius	2	2	20	0.917 ± 0.008	0.547 ± 0.010		
Biceps brachii	1	1	6	0.770 ± 0.026	0.124 ± 0.028		
Brachialis	1	1	16	0.595 ± 0.008	0.797 ± 0.008	0.974 ± 0.009	0.087 ± 0.011
Humeroradialis	1	1	25	0.603 ± 0.004	0.065 ± 0.007		

**FIGURE 3:**
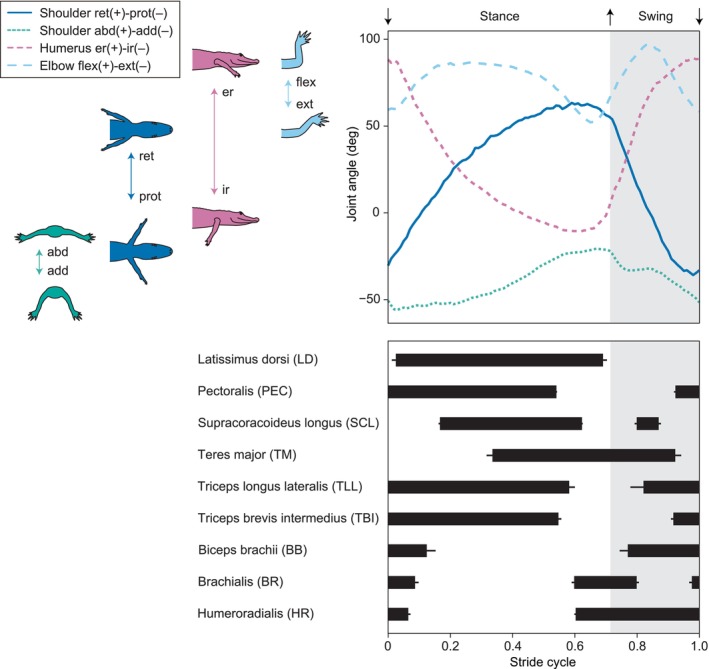
Mean forelimb joint angles of four strides from one representative trial (al13e04) (top), and bar plot showing mean EMG activities for nine forelimb muscles across all recorded trials (bottom). Error bars represent standard error of the mean of onset and offset. Joint angle profiles and muscle onset/offset times were normalized to mean stance and swing ratios across all strides (0.71 stance and 0.29 swing within a stride cycle). Joint movement abbreviations: abd, abduction; add, adduction; er, external rotation; ext, extension; flex, flexion; ir, internal rotation; prot, protraction; ret, retraction.

LD, PEC, SCL, TLL, and TBI exhibited their activities primarily during the stance phase (Figures 2 and 3). LD was active from early through late stance, with its peak occurring at mid‐stance, as the humerus is retracted. PEC was active from late swing through late stance, maintaining a constant level of activation throughout the burst, as the shoulder experiences increased abduction. SCL showed biphasic bursts, with a large primary burst occurring from early through late stance and a small secondary burst occurring at mid‐swing. TLL and TBI showed a similar activation from mid‐swing through late stance, with a discontinuous series of pulses within a single burst. Their activation ends as the elbow starts extending back from the most flexed posture in late stance. TM was active from mid‐stance through late swing, with higher levels of activation observed in late stance and the earliest swing.

BB, BR, and HR exhibited their activities primarily during the swing phase (Figures 2 and 3). BB was active from the earliest swing to early stance, with its peak occurring across the swing‐stance transition. BR showed biphasic bursts, with a higher level of activation at stance‐swing transition and a lower level of activation at the swing‐stance transition. HR was active from late stance, across swing, through early stance.

**FIGURE 4:**
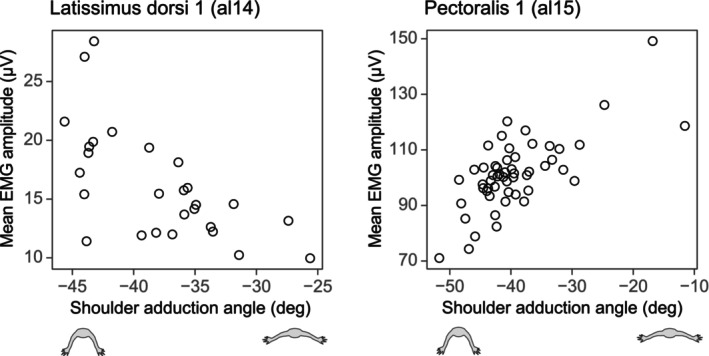
Bivariate plots of mean EMG amplitude and shoulder adduction angle for m. latissimus dorsi 1 in al14 (left) and m. pectoralis 1 in al15 (right).

Multiple linear regressions showed effects of shoulder adduction angle and dimensionless speed on mean EMG amplitude and normalized burst duration for forelimb muscles (Table 2; Figures 4 and S1). Overall, the interaction between shoulder adduction angle and dimensionless speed was nonsignificant for most muscles (Table 2). Significant or near significant interactions between two predictors were observed only in models predicting mean EMG amplitudes of PEC1 (*p* = 0.069), TBI1 (*p* = 0.058), and TBI2 (*p* = 0.030). However, for two TBI channels, small sample sizes (*n* = 7 and 13) and the presence of both positive and negative coefficients for the interaction terms made interpretations challenging. Shoulder adduction angle had a significant effect (*p* < 0.05) on mean EMG amplitude for LD1, PEC1, PEC2, PEC3, and TBI1 (Table 2). Among these muscles, all three channels for PEC showed a positive effect, LD1 showed a negative effect, and one TBI1 showed a negative effect on mean EMG amplitude. Shoulder adduction angle did not exhibit a significant effect on normalized burst duration (Table 2).

**TABLE 2:** Multiple linear regressions predicting EMG variables from shoulder adduction angle (°), dimensionless speed, and their interaction.

Var ~ shoulder adduction angle*dimensionless speed	Number of strides	Shoulder adduction angle coef	Shoulder adduction angle *p*	Dimensionless speed coef	Dimensionless speed *p*	Interaction term coef	Interaction term *p*
Mean EMG amplitude (μV)							
Latissimus dorsi 1 (al14)	27	−0.530	0.002*	15.517	0.415	−1.804	0.587
Latissimus dorsi 2 (al15)	30	0.127	0.638	−72.807	0.011*	4.921	0.421
Pectoralis 1 (al13)	12	0.180	0.030*	90.837	0.019*	14.619	0.069
Pectoralis 2 (al15)	53	1.339	<0.001*	101.293	0.006*	6.452	0.360
Pectoralis 3 (al15)	53	1.890	<0.001*	−83.023	0.081	6.926	0.453
Supracoracoideus longus 1 (al15)	50	0.089	0.817	84.955	0.062	−13.060	0.156
Supracoracoideus longus 2 (al15)	50	0.091	0.800	−12.643	0.760	−11.712	0.171
Teres major (al15)	17	0.051	0.744	74.371	<0.001*	−1.101	0.712
Triceps longus lateralis (al13)	7	0.266	0.775	−1477.403	0.001*	−88.571	0.186
Triceps brevis intermedius 1 (al13)	7	−4.733	0.039*	−2.662	0.985	267.796	0.058
Triceps brevis intermedius 2 (al14)	13	0.361	0.172	−348.281	0.103	−102.652	0.030*
Brachialis (al13)	16	0.549	0.216	63.645	0.319	2.452	0.890
Humeroradialis (al14)	25	−0.322	0.276	246.272	0.006*	−2.412	0.839
Normalized burst duration							
Latissimus dorsi 1 (al14)	27	0.265·10^−2^	0.630	0.104	0.879	−0.064	0.592
Latissimus dorsi 2 (al15)	28	0.118·10^−2^	0.274	0.167	0.112	0.040	0.108
Pectoralis 1 (al13)	12	0.002·10^−2^	0.994	2.359	0.134	−0.434	0.208
Pectoralis 2 (al15)	52	−0.040·10^−2^	0.608	−0.298	0.078	−0.044	0.167
Pectoralis 3 (al15)	52	−0.127·10^−2^	0.094	−0.012	0.939	−0.053	0.083
Supracoracoideus longus 1 (al15)	50	0.177·10^−2^	0.318	0.241	0.241	0.028	0.500
Supracoracoideus longus 2 (al15)	50	0.083·10^−2^	0.557	0.136	0.405	0.038	0.256
Teres major (al15)	17	−0.897·10^−2^	0.097	1.560	0.005*	−0.061	0.537
Triceps longus lateralis (al13)	6	0.306·10^−2^	0.615	−3.984	0.023*	−0.124	0.716
Triceps brevis intermedius 1 (al13)	6	−1.331·10^−2^	0.451	−3.190	0.166	−0.415	0.690
Triceps brevis intermedius 2 (al14)	13	−0.474·10^−2^	0.148	−1.310	0.593	0.305	0.551
Brachialis (al13)	13	−0.236·10^−2^	0.124	0.262	0.256	0.090	0.159
Humeroradialis (al14)	20	0.227·10^−2^	0.248	0.232	0.681	0.085	0.387

*Note*: Predictors were centered around their means prior to the analysis. For muscles characterized by biphasic bursts, the primary burst with higher amplitude and longer duration was used for the analysis. Asterisks indicate significant results (*p* < 0.05).

Abbreviation: coef, coefficient.

**FIGURE 5:**
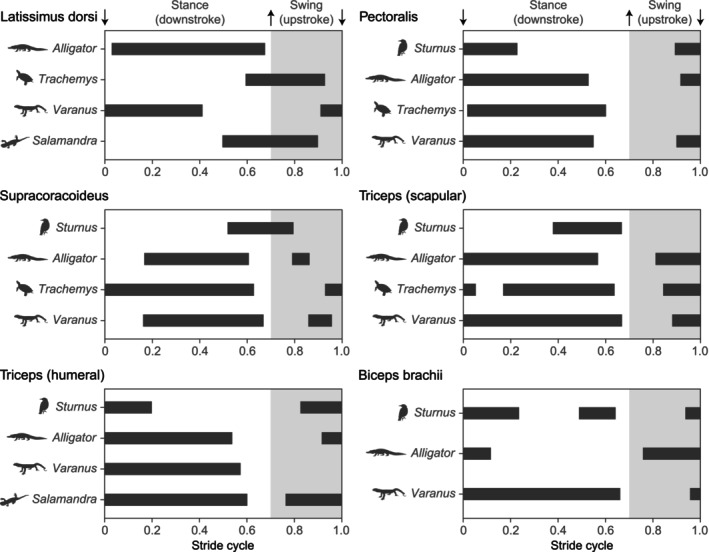
Comparison of forelimb EMG activities among five tetrapods. Muscle onset/offset times were arbitrarily normalized to 0.7 stance (upstroke) and 0.3 swing (downstroke) within a stride (stroke) cycle. Animal silhouettes from phylopic.org (uploaded by Maxime Dahirel, Ferran Sayol, Brian O'Meara, Christina Zdenek, and Beth Reinke).

**FIGURE 6:**
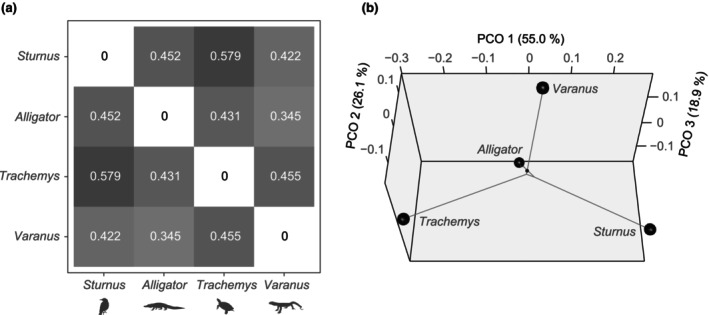
Quantitative comparisons of forelimb EMG activities among tetrapods. (a) EMG burst phase distance matrix based on six homologous forelimb muscles from four sauropsids and (b) ‘phylo‐EMG space’ generated from the distance matrix. A small black dot connected to the branch from *Varanus* represents the ancestral node of the four taxa. Animal silhouettes from phylopic.org (uploaded by Maxime Dahirel, Ferran Sayol, Brian O'Meara, and Christina Zdenek).

Comparisons of normalized forelimb EMG activities among five tetrapods (*Sturnus*, *Alligator*, *Trachemys*, *Varanus*, and *Salamandra*) revealed varying degrees of similarity in muscle activation patterns across taxa (Figure 5). Muscle activation timings showed some similarities for m. pectoralis and m. triceps (humeral heads), but exhibited greater variability for m. latissimus dorsi, m. supracoracoideus, m. triceps (scapular head), and m. biceps brachii among the five taxa. Particularly, significant differences in EMG burst phase were found for m. latissimus dorsi between *Alligator* and *Varanus* compared to *Trachemys* and *Salamandra*, for m. supracoracoideus between *Sturnus* and non‐avian sauropsids, and for m. biceps brachii between *Sturnus* and *Varanus* compared to *Alligator*. An EMG burst phase distance matrix based on six forelimb muscles from four sauropsids (*Sturnus*, *Alligator*, *Trachemys*, and *Varanus*) showed that pairwise distances are shortest (0.345) between *Alligator* and *Varanus*, and longest (0.579) between *Sturnus* and *Trachemys* (Figure 6).

## 4 DISCUSSION

### 4.1 Effect of limb posture on forelimb muscle activation

One perceived benefit of assuming a more adducted shoulder in sprawling taxa is the alignment of the forelimb with the ground reaction force, which helps to reduce limb muscle force and work. Consistent with this expectation, mean EMG burst amplitudes of PEC, a primary shoulder adductor, were significantly smaller when walking with a more adducted shoulder, despite similar normalized burst durations across different limb postures (Table 2; Figure 4). This relationship appears to be robust, as it was observed in all three PEC electrode channels from two animals. PEC is the largest shoulder muscle in *Alligator* and the combined muscle mass from both forelimbs constitutes ~1.38% of total body mass (Allen et al., 2010). Consequently, the modulation of forelimb posture would result in a significant reduction in net forelimb force and work.

Alternatively, the correlation between EMG amplitudes and limb posture may suggest that PEC operates at a less optimal fiber length, requiring greater recruitment of the muscle when walking with a more abducted shoulder. However, it is important to note that muscle fibers typically operate on the ascending limb and plateau of the force–length curve (Arnold & Delp, 2011; Bishop et al., 2021; Rubenson et al., 2012). In the case of PEC muscle fibers, when the humerus is abducted, they would be more stretched and operate near the plateau of the force–length curve. Assuming constant forces, this would result in reduced activation of PEC during an abducted limb walk, unlike the pattern observed in the current study. Other confounding factors, such as force‐velocity properties and muscle fiber types, require further examination to understand their interaction with muscle recruitment.

Our findings for PEC activity align with previous research investigating the effect of limb posture on EMG amplitude in mammals. Studies on humans and cats showed that a more flexed hindlimb posture leads to higher EMG amplitude not only in antigravity muscles, such as knee extensors and ankle plantarflexors, but also in non‐antigravity muscles, such as knee flexors and ankle dorsiflexors (Grasso et al., 2000; Trank et al., 1996). This can be attributed to increased limb joint moments imposed by the ground reaction force and reduced energy recovery through pendular mechanisms during a crouched limb walk, which increase mechanical work during both stance and swing (Grasso et al., 2000; Trank et al., 1996). Concordant with the EMG data, 3D musculoskeletal modeling of human crouched walking has demonstrated an increase in the average force exerted by stance limb muscles compared to the force during normal walking, due to smaller body weight support from the limb skeleton and a reduced capacity to extend the limb joints (Hicks et al., 2008; Steele et al., 2010).

Contrary to findings from mammals, the crocodylian hindlimb showed an increase in mean EMG amplitude for most of stance and swing limb muscles when the hip was in a more adducted position (Reilly & Blob, 2003). To explain this counterintuitive trend, the chain muscle counteraction hypothesis has been proposed (Blob & Biewener, 2001; Reilly & Blob, 2003). According to this hypothesis, there is an anterior shift in the center of pressure (CoP) of the hindlimb during an upright walk. This shift leads to larger ankle plantarflexor forces to counteract the increased ankle joint moment. Because one of the primary ankle plantarflexors, m. gastrocnemius, is a biarticular muscle spanning across both the ankle and knee joints and is involved in knee flexion, knee extensor forces are also expected to increase. In accordance with the supposedly higher knee extensor forces, compressive strains on the dorsal femoral cortex and tensile strains on the ventral femoral cortex increase during an adducted limb walk (Blob & Biewener, 1999). The increased activation of hindlimb muscles during an upright walk may also be related to the increased load on the hindlimb to support the elevated tail, which constitutes 20%–28% of the total body mass (Iijima et al., 2023; Willey et al., 2004). However, the chain muscle counteraction mechanism may not be applicable to the forelimb of alligators due to the shorter length of the manus relative to the pes, and a limited range of motion for wrist dorsiflexion (Hutson & Hutson, 2014). Additionally, tail elevation might impact loads on the hindlimb to a greater extent than loads on the forelimb. To better understand the limb‐specific interactions between posture and motor patterns in the forelimb and hindlimb, integrated data on limb kinematics, external forces, motor patterns, and bone strains (Reilly et al., 2005) from both limbs would be required.

The increased activation of the shoulder adductor muscle when walking with an abducted limb is also consistent with the size‐dependent changes in limb posture observed in American alligators. As mass‐specific demands for both force and work increase in proportion to mass^1/3^ under geometric and dynamic similarities, larger mammals and birds are known to change gait, limb posture, and body shape to reduce mechanical demands (Biewener, 1989, 1990, 2005, 2015; Daley & Birn‐Jeffery, 2018; Gatesy & Biewener, 1991). Locomotor experiments on American alligators ranging in size from 0.2 to 223 kg showed that individuals with body mass of over 1 kg assume more adducted shoulder and hip postures (Iijima et al., 2021, 2023). Because shoulder and hip abduction moments were typically smaller when walking with adducted limbs, the use of more adducted limbs in larger alligators might be a strategy to mitigate mechanical demands on shoulder and hip adduction muscles (Iijima et al., 2021). If the continuum of abducted‐adducted limb postures in alligators is size‐dependent as in the continuum of crouched to extended limb postures in mammals and birds (Biewener, 1989; Daley & Birn‐Jeffery, 2018), sprawling to erect (abducted to adducted) postural transitions could be either the cause or consequence of the evolution of larger body size in archosaurs. Trackway evidence suggests that proxies of limb posture and body size (i.e., pace angulation and foot length) were not correlated during the Permian and Triassic, time periods across which independent lineages of tetrapods experienced shifts toward more erect limb postures (Kubo & Benton, 2009). However, the acquisition of a more erect limb posture in the Early Triassic, along with an increase in the upper limit of body size throughout the Triassic and Jurassic in archosauromorphs (Kubo & Benton, 2009; Kubo & Kubo, 2016; Sookias et al., 2012; Turner & Nesbitt, 2013), suggests that a transition to more upright posture may have facilitated the evolution of larger body size in archosauromorphs.

Excluding the trend observed for PEC, one of the electrodes placed in each of LD and TBI (LD1 and TBI1) showed increased mean EMG amplitudes with a more adducted limb posture (Table 2; Figure S1). Although the model predicting mean TBI1 amplitude is hard to interpret due to the small sample size (*n* = 7), small range of humerus adduction angle (−43° ~ −37°), and the interaction between two predictors, the trend for LD1 appears to be relatively strong (Table 2; Figure 4). LD is a stance phase humerus retractor, originating from the anterior trunk, passing posterior to the caudal edge of the scapula, and inserting on the craniodorsal surface of the proximal humerus (Meers, 2003). Because of this muscle configuration, the moment arm for shoulder extension in LD would be reduced during a more adducted limb walk, requiring larger forces to retract the humerus during stance. However, the mass of LD from both forelimbs constitutes only 0.164% of the total body mass (Allen et al., 2010), thus its effect on net muscle force and work would be smaller compared to that of PEC. Among other antigravity muscles, TLL, a major elbow extensor during stance, did not show modulation in its activation level across different shoulder adduction angles (Table 2). This could be attributed to the smaller sample size (*n* = 7) and smaller range of humerus adduction angle (−44° ~ −36°). Alternatively, it could mean that the elbow flexion moment caused by external forces remains consistent regardless of shoulder adduction angles.

### 4.2 Variation in forelimb muscle activation patterns among tetrapods

We have provided EMG data for nine forelimb muscles from American alligators during terrestrial locomotion, significantly expanding the coverage of EMG recordings in crocodylians that are the closest extant relatives of birds. Previous studies have argued for an overall similarity in activation patterns for homologous forelimb muscles, with some variation related to differences in locomotor modes (e.g., aquatic, terrestrial, and aerial locomotion: Cuff et al., 2019; Dial et al., 1991; Jenkins & Goslow, 1983; Pierce et al., 2020; Rivera & Blob, 2010). The comparisons of activation patterns among six homologous forelimb muscles presented here have contributed to a more detailed understanding of the interplay of phylogenetic inertia and adaptation in the evolution of forelimb motor patterns, particularly in relation to the acquisition of powered flight in birds (Figure 5).

Antigravity muscles including m. pectoralis and mm. triceps (scapular and humeral heads) show similar activation patterns among non‐avian tetrapods (*Alligator*, *Trachemys*, *Varanus*, and *Salamandra*) during terrestrial locomotion, while they were altered in birds (*Sturnus*) during flight (Jenkins & Goslow, 1983; Pierce et al., 2020; Rivera & Blob, 2010: Figure 5). In non‐avian tetrapods, m. pectoralis and mm. triceps are typically activated from mid‐swing through late stance, generating forces to support the body during stance. However, in *Sturnus*, the offset of m. pectoralis is shifted earlier, occurring within the first half of the downstroke during flight. Furthermore, the functions of mm. triceps, which no longer play an antigravity support role, differ in *Sturnus*. Specifically, m. humerotriceps serves as an elbow extensor from the mid‐upstroke through the early downstroke, and m. scapulotriceps serves as an elbow stabilizer during the latter half of the downstroke in *Sturnus* (Dial et al., 1991).

M. supracoracoideus is another muscle that has undergone functional changes through the acquisition of powered flight in birds (Figure 5). The activation patterns of m. supracoracoideus in *Alligator* and *Varanus* are similar, exhibiting a large primary burst during stance and a small secondary burst during swing to stabilize the glenohumeral joint (Jenkins & Goslow, 1983). Although monophasic, *Trachemys* also exhibits activation of m. supracoracoideus throughout stance, but its function is regarded as humerus retraction and depression (Rivera & Blob, 2010). However, in *Sturnus*, m. supracoracoideus is active from late downstroke through early upstroke, functioning as a primary humeral elevator and supinator (Biewener, 2011; Dial et al., 1991; Poore et al., 1997).

Other muscles show diverse activation patterns both within the muscle and across different taxa (Figure 5). M. latissimus dorsi is active throughout stance in *Alligator* as a humeral retractor, while it is active from late stance to late swing in *Trachemys* and *Salamandra* as a humeral elevator (Pierce et al., 2020; Rivera & Blob, 2010). Its activation differs across different parts of the muscle in *Varanus*: middle and posterior parts are active from late swing through late stance as a humeral retractor, whereas the anterior part adds an additional burst from late stance through mid‐swing as a humeral elevator (Jenkins & Goslow, 1983). Although a comprehensive assessment of within‐muscle activation patterns is required across tetrapods, it appears that the functions of stance phase humeral retraction and swing phase humeral elevation are retained among non‐avian tetrapods. M. biceps brachii is active from late swing through late stance as a glenohumeral and elbow stabilizer in *Varanus* (Jenkins & Goslow, 1983), while it is active from early swing through early stance as an elbow flexor in *Alligator*. In *Sturnus*, the activation of m. biceps brachii is biphasic, across the upstroke‐downstroke transition to protract the humerus and decelerate elbow extension, and during late downstroke to flex the elbow (Dial et al., 1991).

A ‘phylo‐EMG space’ based on six homologous forelimb muscles from four sauropsids (*Sturnus*, *Alligator*, *Trachemys*, and *Varanus*) revealed a resemblance in the EMG burst phases between *Alligator* and *Varanus*, which share a similar body plan and limb kinematics compared to the other taxa (Baier & Gatesy, 2013; Blob & Biewener, 2001; Jenkins & Goslow, 1983; Reilly & Elias, 1998: Figure 6b and Movie S1). By contrast, *Sturnus* and *Trachemys*, which have undergone major musculoskeletal transformations such as the development of the wings and bony shell, displayed more distinct burst phases (Figure 6b and Movie S1). However, it should be noted that the implication of this quantitative comparison may be limited due to the small numbers of muscles and taxa included in the analysis. The current comparison also did not consider temporal changes in burst amplitude within a burst, or variation in burst phases across different regions of a muscle. Normalization of stance and swing times in terrestrial sauropsids and downstroke and upstroke times in flying birds to a uniform ratio presents a further caveat for comparisons, as this could induce confounding factors when comparing burst phases. Nonetheless, our results support the notion that forelimb muscle activation patterns were influenced by major changes in body plan and locomotor modes, regardless of phylogenetic relatedness, in tetrapods leading to archosaurs. Such an interpretation is consistent with observed intraspecific variation in motor patterns across different locomotor modes and habitats in tetrapods, reinforcing conclusions about the extent to which muscle function can vary in relation to differing demands (Blob et al., 2008; Foster & Higham, 2014; Gillis & Blob, 2001; Gorvet et al., 2020; Rivera & Blob, 2010).

We acknowledge the limitations of the current study. First, among the nine muscles recorded for their activities, five were from a single electrode, and six were from a single individual. This posed challenges in considering the effect of intraspecific variation in muscle activation patterns, particularly regarding the activation timings. Indeed, when comparing activities of a single muscle across multiple electrode channels and individuals, variations in normalized burst timings were observed for each muscle (Table S1). Second, in relation to the first limitation, regional variation in activation patterns within a single muscle was not considered, except for a few instances (Figure 1). Previous studies suggested variations in activation timings across distinct regions within a single muscle, particularly evident in those with broad bellies, including m. latissimus dorsi and m. pectoralis (Jenkins & Goslow, 1983; Jenkins & Weijs, 1979). The low sample size of the current study is partly due to the low successful rate (39%) of EMG recording in our alligator experiments. This is attributed to various challenges such as behaviors involving shoulder and neck scratching with hindlimbs, often resulting in the loss of a bundle of electrode channels. Despite these limitations, given the phylogenetic position of crocodylians bracketed by lepidosaurs and birds within saurians, where major locomotor transitions occurred, our study provides crucial data regarding changes in forelimb muscle function across limb postures and locomotor modes.

## 5 CONCLUSION

This study described EMG activities of nine forelimb muscles in American alligators, with the aim of understanding changes in forelimb muscle activations across limb postures and taxa to gain insights into major locomotor transitions. EMG amplitudes of m. pectoralis, a primary shoulder adductor, were lower when walking with a more adducted forelimb, potentially resulting in a reduced net forelimb force and work during stance. Among sauropsids, forelimb muscle burst phases from birds and turtles are distinct compared to those of alligators and lizards, suggesting that muscle activation patterns were influenced by major changes in body plan and locomotor modes.

## AUTHOR CONTRIBUTIONS

Masaya Iijima designed and conducted the experiments, wrote the R script, analyzed the data, created the figures, and drafted the manuscript. Christopher J. Mayerl wrote the original script for EMG signal thresholding and on/off time determination and edited the manuscript. V. David Munteanu conducted the experiments and edited the manuscript. Richard W. Blob supervised the project, designed and conducted the experiments, and helped draft the manuscript.

## FUNDING INFORMATION

JSPS Grant‐in‐Aid for Scientific Research, Grant/Award Number: 19J00701.

## CONFLICT OF INTEREST STATEMENT

The authors declare no competing financial, professional, or personal interests.

## Supporting information


**Data S1.**.

## Data Availability

R script and metadata used to perform analyses are available from the figshare repository: https://doi.org/10.6084/m9.figshare.24530404.
