# Supplementary material for: Forelimb muscle activation patterns in American alligators: Insights into the evolution of limb posture and powered flight in archosaurs
Source: J Anat. 2024 Jan 19;244(6):943–58. doi: 10.1111/joa.14011 (PMC11095314; doi:10.1111/joa.14011)

**TABLE S1** Summary of forelimb EMG onset and offset (mean ± standard error) normalized to a relative stride time ranging from 0 to 1, for individual electrode channels and animals.

| Muscle | Animal | Burst 1 onset | Burst 1 offset | Burst 2 onset | Burst 2 offset |
| --- | --- | --- | --- | --- | --- |
| Latissimus dorsi 1 | al14 | 0.068±0.024 | 0.615±0.019 |  |  |
| Latissimus dorsi 2 | al15 | 0.985±0.004 | 0.760±0.002 |  |  |
| Pectoralis 1 | al13 | 0.999±0.004 | 0.540±0.009 |  |  |
| Pectoralis 2 | al15 | 0.879±0.004 | 0.536±0.004 |  |  |
| Pectoralis 3 | al15 | 0.948±0.004 | 0.545±0.004 |  |  |
| Supracoracoideus longus 1 | al15 | 0.168±0.007 | 0.629±0.005 | 0.798±0.010 | 0.859±0.012 |
| Supracoracoideus longus 2 | al15 | 0.165±0.006 | 0.616±0.003 | 0.800±0.009 | 0.877±0.010 |
| Triceps brevis intermedius 1 | al13 | 0.949±0.016 | 0.557±0.011 |  |  |
| Triceps brevis intermedius 2 | al14 | 0.902±0.006 | 0.542±0.014 |  |  |

**FIGURE S1** Bivariate plots of mean EMG amplitude and shoulder adduction angle for m. pectoralis 1 (al13), m. pectoralis 2 (al15), and m. triceps brevis intermedius 1 (al13).


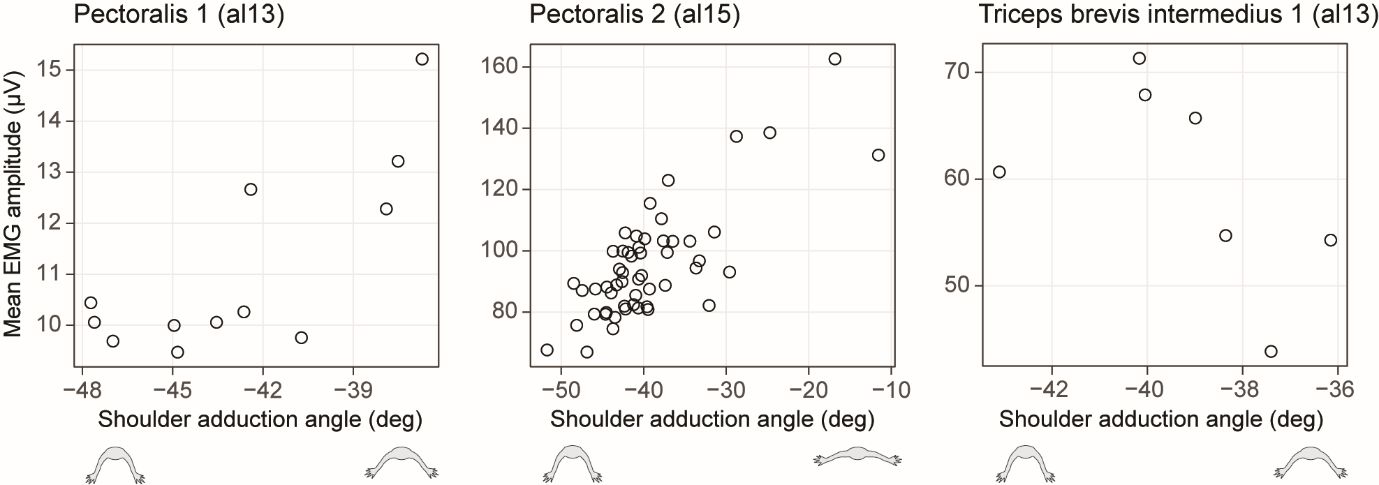


**MOVIE S1** 3D ‘phylo-EMG space’ generated from EMG burst phase distance matrix based on six homologous forelimb muscles from four sauropsids. Species abbreviations: AM, *Alligator mississippiensis*; SV, *Sturnus vulgaris*; TS, *Trachemys scripta*; VE, *Varanus* *exanthematicus*.


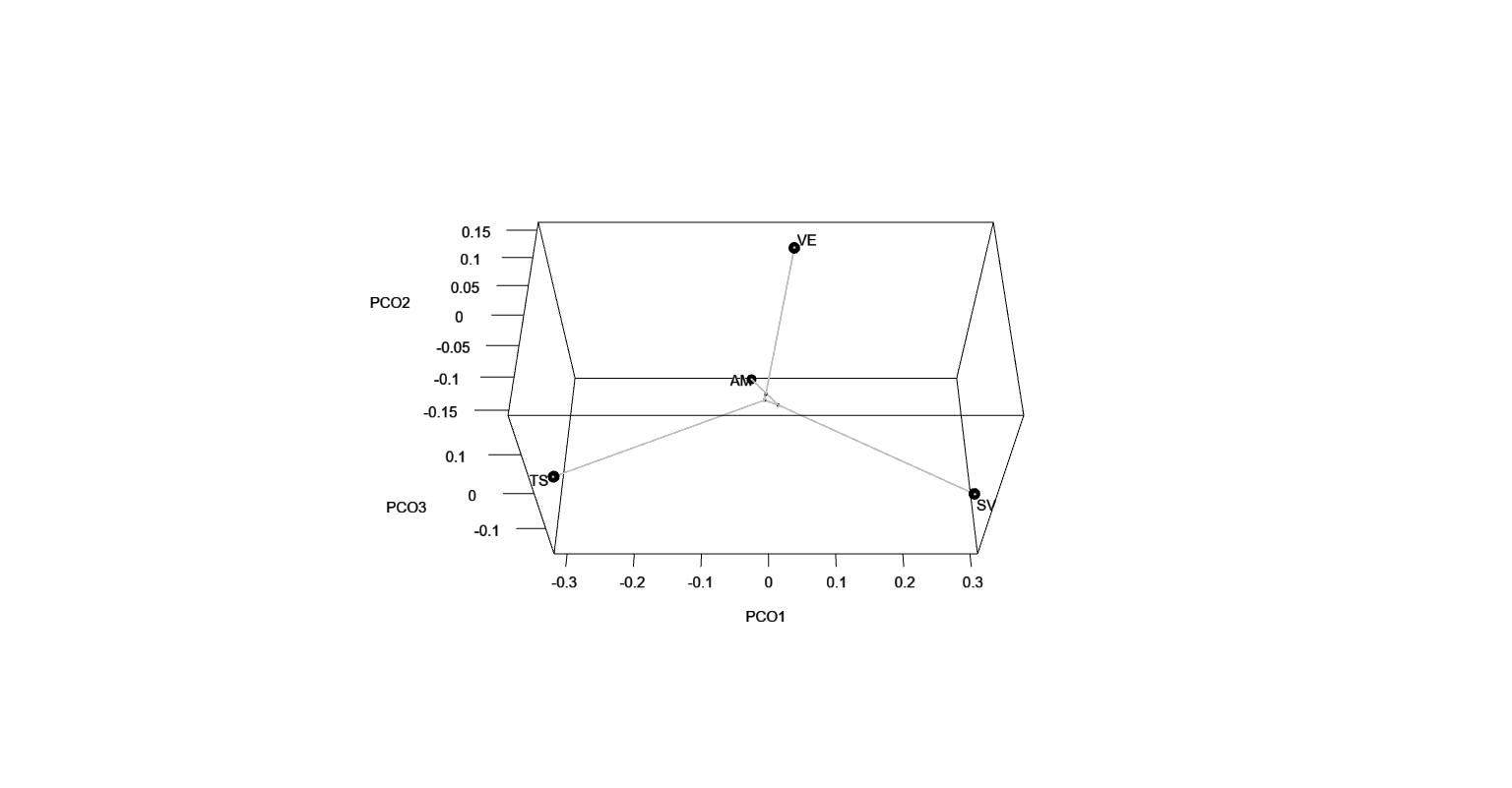

Supplement: Supplementary file 1 — Data S1.. [file JOA-244-943-s001.docx]
